# Design, Synthesis and Biological Evaluation of New Piperazin-4-yl-(acetyl-thiazolidine-2,4-dione) Norfloxacin Analogues as Antimicrobial Agents

**DOI:** 10.3390/molecules24213959

**Published:** 2019-10-31

**Authors:** Gabriel Marc, Cătălin Araniciu, Smaranda Dafina Oniga, Laurian Vlase, Adrian Pîrnău, George Cosmin Nadăș, Cristiana Ștefania Novac, Ioana Adriana Matei, Mariana Carmen Chifiriuc, Luminița Măruțescu, Ovidiu Oniga

**Affiliations:** 1 Faculty of Pharmacy, “Iuliu Hațieganu” University of Medicine and Pharmacy, 8 Victor Babes Street, 400012 Cluj-Napoca, Romania; Marc.Gabriel@umfcluj.ro (G.M.); laurian.vlase@umfcluj.ro (L.V.); ooniga@umfcluj.ro (O.O.); 2 National Institute for Research and Development of Isotopic and Molecular Technologies, 67-103 Donath Street, 400293 Cluj-Napoca, Romania; adrian.pirnau@itim-cj.ro; 3 Department of Microbiology, Faculty of Veterinary Medicine, University of Agricultural Sciences and Veterinary Medicine, 3-5 Mănăștur Street, 400372 Cluj-Napoca, Romania; gnadas@usamvcluj.ro (G.C.N.); matei.ioana@usamvcluj.ro (I.A.M.); 4 Department of Microbiology, Faculty of Biology, University of Bucharest, 1-3 Portocalelor Street, 60101 Bucharest, Romania; carmen_balotescu@yahoo.com (M.C.C.); lumi.marutescu@gmail.com (L.M.); 5 Research Institute of the University of Bucharest-ICUB, 91-95 Independentei Street, 050095 Bucharest, Romania

**Keywords:** norfloxacin, thiazolidinedione, anti-biofilm, anti-bacterial, DNA gyrase, antibiotic resistance, fluoroquinolones

## Abstract

In an effort to improve the antimicrobial activity of norfloxacin, a series of hybrid norfloxacin–thiazolidinedione molecules were synthesized and screened for their direct antimicrobial activity and their anti-biofilm properties. The new hybrids were intended to have a new binding mode to DNA gyrase, that will allow for a more potent antibacterial effect, and for activity against current quinolone-resistant bacterial strains. Moreover, the thiazolidinedione moiety aimed to include additional anti-pathogenicity by preventing biofilm formation. The resulting compounds showed promising direct activity against Gram-negative strains, and anti-biofilm activity against Gram-positive strains. Docking studies and ADMET were also used in order to explain the biological properties and revealed some potential advantages over the parent molecule norfloxacin.

## 1. Introduction

Introduced into therapy in the early 60s, quinolones have rapidly become one of the most used classes of antibacterial agents. They are very appreciated because of their ability to treat a wide variety of infections (urinary tract, respiratory, skin, soft tissue, sexually transmitted diseases and even bone infections) caused by some of the most common pathogens [1]. As a consequence of their success, drug discovery and development yielded many new compounds, but the wide-scale use of these drugs lead to an increase of bacterial resistance, as observed with most antibacterial agents.

Currently, developing new fluoroquinolones should focus on finding molecules that overcome resistance, while maintain a broad antibacterial activity [2]. The key elements in this process must be the understanding of quinolone molecular mechanism of action, via the topoisomerase mechanism, and also discerning the main mechanisms of bacterial resistance to quinolone derivatives [3,4].

Fluoroquinolones’ mechanism of action consists of inhibition of two enzymes—DNA gyrase and DNA topoisomerase IV—that are crucial for DNA replication, thus inhibiting bacterial growth and causing cell death [1]. The topoisomerase IV bacterial enzyme is the main target of fluoroquinolones in Gram-positive bacteria. Fluoroquinolones have a 1000-fold selectivity towards this bacterial enzyme, compared with the human one. In Gram-negative bacteria, the key target of fluoroquinolones is DNA gyrase [1,4]. DNA gyrase and DNA topoisomerase IV are both classified as type IIA topoisomerases and are responsible for controlling bacterial DNA topology by interconverting relaxed and supercoiled forms of DNA [3,4,5]. The two enzymes have similar activities, with the notable difference that DNA gyrase is also capable of introducing negative supercoiling [6]. Structurally, DNA gyrases consists of two GyrA subunits and two GyrB subunits, resulting in a heterotetrameric structure (A_2_B_2_) [4,6]. The GyrA subunit is responsible for non-catalytic interactions with DNA, while the GyrB effectively catalysis the DNA strands cutting and rejoining. In the case of topoisomerase IV, the corresponding subunits are called ParC and ParE [5].

The analysis of the intimate molecular interactions between fluoroquinolones and their targets revealed that fluoroquinolones inhibit DNA gyrase and topoisomerase IV by forming a ternary complex comprised of the drug, the enzyme and bound DNA. This is possible because the binding site of the fluoroquinolones is exposed only once the DNA has been bounded to the enzyme, split and is ready to be crossed over. The key structural elements of the fluoroquinolones’ scaffold are represented by the carbonyl and carboxylate group (C-3, C-4) which interact with DNA, the 6 fluoro substituent, the C-7 position substituent and the carboxylate ion which form binding interactions with the enzyme [7]. The ternary complex is formed due to the fact that fluoroquinolones are able to bind to the topoisomerase-DNA covalent complex via a Mg^2+^ -water-bridge between the C-3, C-4 diketo moiety and some conserved residues found in GyrA/ParC (Ser-83, Asp-87 / Ser-84, Glu-88) [5,8]. In some cases, the quinolones are also capable of another interaction between the C-7 ring systems and the GyrB/ParE region of the enzyme [5,6,9].

The second important aspect in developing new fluoroquinolones is the variety of mechanisms that lead to antibiotic resistance. These include: permeability-based resistance (a deficiency of particular porin proteins that normally serve as channels for the hydrophobic quinolones, or other alterations of the lipopolysaccharide from the cell envelope membranes that prevent the bacterial uptake of quinolone) or efflux-based fluoroquinolone resistance (due to an over-expression of genes involved in the removal of harmful agents from the bacterial cell) [3,10]. Another important resistance mechanism is the one based on target-enzyme modification. This is caused by mutations of the gyrase genes (*gyrA, gyrB*) or the topoisomerase IV genes (*parC*, *parE*) that change the amino acid sequences of the fluoroquinolones’ target enzymes. The most frequent mutations that cause resistance are linked with the modification at the Ser-83 and Asp-87 residues present in the *E. coli* GyrA. These residues correspond to Ser-84 and Glu-88 found in the ParC subunit of topoisomerase IV [3,8]. Moreover, other significant factors in the acquisition of fluoroquinolone resistance are their ability to induce a mutagenic SOS response and the presence of plasmid-mediated resistance [3].

As result of the fact that most resistance is caused by mutation in the Ser-83 and Asp-87 residues of GyrA, recent efforts are focusing on obtain analogs that are capable of having extra-interactions with the target, in a manner that makes the magnesium-water-bridge less crucial. By bypassing the need for an interaction with residues that are easily mutated, these compounds could be active against current quinolone-resistant bacterial strains. Moreover, because they are able to simultaneously interact with different parts of the enzyme, they could form more stable complexes with DNA-topoisomerases and thus be more potent [8,9,11].

An overview of the structural modulations performed on quinolones, as described in literature reviews, suggest that the most successful approach in obtaining new quinolones is maintaining the main scaffold and obtaining analogs via molecular hybridization [2,12,13,14,15,16,17,18]. Most fluoroquinolone analogs are obtained by modulating the C-7 position with various substituents or ring systems (azoles, furan, thiophene, indole, anilinouracil) [15]. One such example is cadazolid, an antibiotic that combines the scaffold of fluoroquinolone with an oxazolidinone pharmacophore [19]. This new drug class is known as the quinoxolidinones and contains many promising molecules, including MCB3837 and MCB3681 [20]. In this context, our previous efforts [21] represent the first known report of a thiazolidinedione-fluoroquinolone hybridization and as such, we set out to obtain new norfloxacin derivatives that include a thiazolidinedione substituent at the piperazin-4-yl moiety. The key rationale behind choosing thiazolidinedione was the versatility of this nucleus, that is present in many antimicrobial and anti-biofilm molecules [22,23,24,25]. Thus, the new norfloxacin hybrids could act by directly binding to topoisomerase, and in the same time, have an additional effect by inhibition of biofilm formation. Biofilm leads to increased bacterial resistance to antibiotics and also increased bacterial pathogenicity and it is well established that anti-biofilm agents act as potential antipathogens [26,27,28,29]. Moreover, the C-7 multi-ring substituents where chosen with the intent of substantially modifying physicochemical properties (size, charge, lipophilicity) in order to improve the pharmacokinetic profile, alter bacterial cell penetrability affecting permeability-based antibiotic resistance, improve DNA-topoisomerase complex binding mode and possibly gain anti-biofilm activity as an adjuvant mechanism of action.

**Figure 1:**
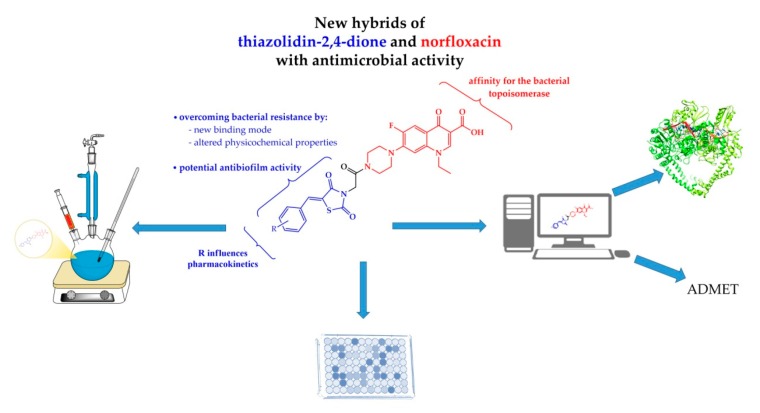
The design rational for the new piperazin-4-yl-(acetyl-thiazolidine-2,4-dione) norfloxacin analogues

Based on this structural rationale we set out to synthesize new molecular hybrids of norfloxacin, we assessed their direct antimicrobial potential, as well as their anti-biofilm activity. In order to provide explanations for the biological evaluation results we performed docking studies using DNA-gyrase. The druggability of the new molecules was also estimated using in silico ADMET tools (Figure 1).

## 2. Results and Discussion

### 2.1. Chemistry

**Scheme 1:**
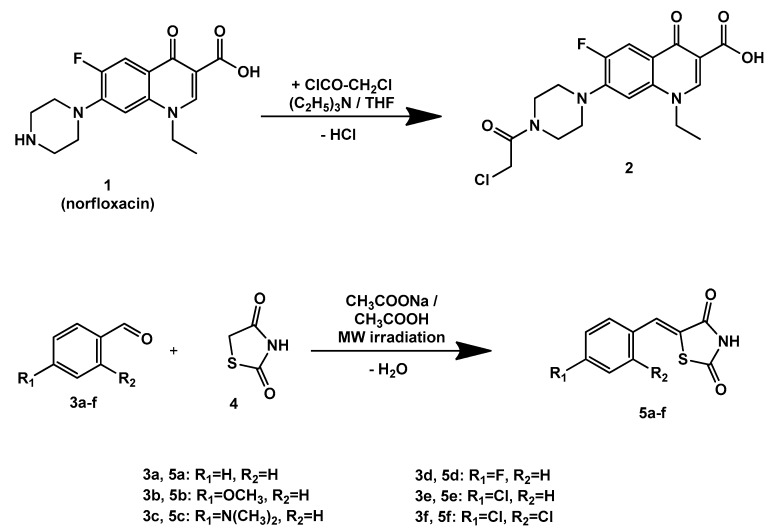
The synthesis scheme of the intermediate compound **2** and (*Z*)-5-benzylidene-thiazolidine-2,4-dione derivatives **5a**–**f**.

**Figure 2:**
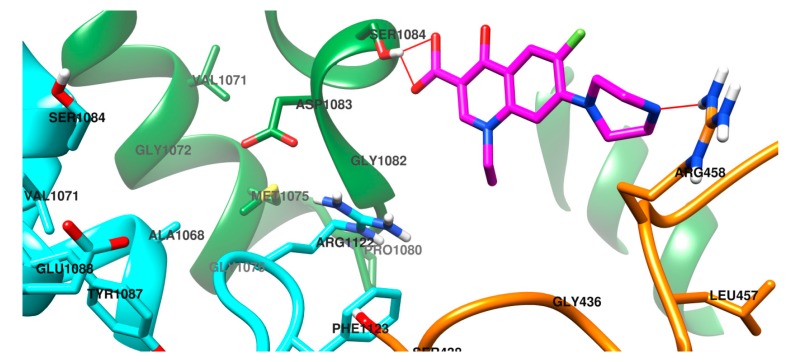
The molecule of norfloxacin docked in the active site of bacterial DNA gyrase. The two subunits of gyrase are depicted in orange (GyrB), respectively green (GyrA). The cross-linking made by norfloxacin between the two subunits is depicted with red lines.

A total of seven new hybrid compounds **6**, **7a**–**f** between norfloxacin and thiazolidine-2,4-dione were synthesized. The chemical linkage between the two fragments was made through an acetamide bridge. First, a chloroacetamide derivative of norfloxacin (compound **2**) was synthesized by treating it with chloroacetyl chloride in tetrahydrofuran (THF) alkalized with triethylamine (TEA), as depicted in Figure 2. In parallel, 5-benzylidene-thiazolidine-2,4-dione derivatives (compounds **5a**–**f**) were synthesized by Knoevenagel condensations of thiazolidine-2,4-dione (compound **4**) with some aromatic aldehydes (compounds **3a**–**f**), under microwave irradiation. The alkaline catalyst required to conduct the Knoevenagel condensation was anhydrous sodium acetate, in acetic acid as solvent (see Scheme 1).

**Scheme 2:**
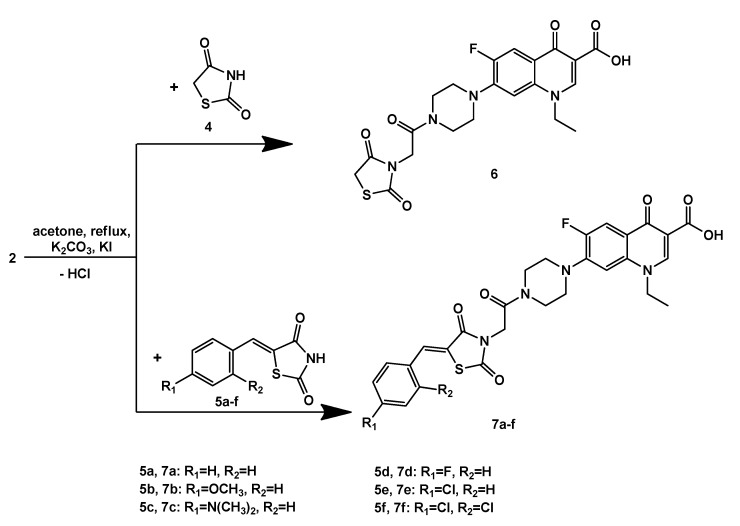
The synthesis scheme of the final compounds **6** and **7a**–**f**.

Finally, the alkylation of thiazolidine-2,4-dione, or of its 5-benzilidene derivatives **5a**–**f**, took place under alkaline conditions provided by anhydrous K_2_CO_3_. using anhydrous acetone as solvent. A Finkelstein transhalogenation reaction was applied to convert in situ the intermediate chlorine-derivative compound **2** into its more reactive iodine-analog [30]. The alkylation reaction was possible due to the acidity of the proton at position 3 of thiazolidine-2,4-dione, which provides, in an alkaline environment, a nucleophilic anionic species responsible for the attack of the halogenated intermediate compound **2** [31], as shown in Scheme 2.

In all final compounds five strong νC=O stretching bonds were found. Stretching of the bonds corresponding to norfloxacin (carboxylic acid and ketone) were found in all compounds between 1725–1737 cm^−1^, respectively 1696–1704 cm^−1^. A new supplementary signal, a stretching of the amide C=O between 1710–1718 cm^−1^, was found in all norfloxacin hybrids. The successful insertion of the thiazolidine-2,4-dione in the molecule of the final compounds was confirmed by the appearance of a supplementary two C=O stretching bands from the thiazolidine-2,4-dione nucleus, between 1748–1760 cm^−1^, respectively 1678–1695 cm^−1^.

The presence of the molecular ions in the MS spectra was found for compounds **7e**,**7f** in our series, with the expected distinctive isotopic pattern.

In all compounds, the protons were found at the expected values and with the expected multiplicity. A strong singlet proton was found between 8.96–8.98 ppm, corresponding to the proton from the 2-position of the quinolone. Protons from positions 5 and 8 of the quinolone were found between 7.98–7.93 ppm and 7.21–7.25 ppm, respectively. Both protons were coupled with the fluorine atom. The four methylene protons from the piperazine ring, next to the quinolone, were found as broad signals between 3.41–3.58 ppm,. The other methylene protons from the piperazine ring, those close to the amide bridge, were found between 3.68–3.80 ppm, also as broad signals. Due to an anisotropic effect, caused by the presence of the large polar substituent introduced on the piperazine nitrogen atom, the protons located in the vicinity of the amide bridge appear different from those close to the quinolone nucleus, as two broad peaks with, corresponding to two protons each.

Concerning the ^13^C-NMR spectra of compounds **2**, **6** and **7a**–**f**, five peaks corresponding to C=O carbon atoms were found between 161.27–177.31 ppm. The carbon atom from the 6 position of quinolone nucleus was found between 153.54–153.29 ppm, with a coupling constant *J* = 247 Hz, due to the fluorine atom linked directly to it. The C–F coupling effect was also found for the carbon atom from the position 5 of quinolone.

### 2.2. Biological Assays

#### 2.2.1. Antimicrobial Activity—Initial In Vitro Qualitative Screening Study

The starting compound (norfloxacin), together with the chloroacetamide intermediate (**2**), and the seven final compounds **6**,**7a**–**f** were subjected to an initial in vitro qualitative screening. Antimicrobial activity was initially evaluated using a total number of seven bacterial and two yeast strains. This initial step aimed to identify the antimicrobial potential of the compounds and to select the newly synthesized derivatives with increased antimicrobial potential. An overall increased efficiency was observed against Gram-negative bacteria, compared to Gram-positive strains, as seen in Table 1. The diameters of the inhibition areas for Gram-negative species ranged from 14 to 32 mm, while for Gram-positive strains it was between 8 and 23 mm.

It is well known that norfloxacin is mostly active against Gram-negative bacteria, while derivatives that possess an extra halogen or methoxy moiety in the C-8 position and varied C-7 ring systems (sparfloxacin, moxifloxacin) have improved Gram-positive activity [3,6]. The results of this primary assessment let us to assume that the new hybrids, which are not substituted at the C-8 position, maintain the profile of norfloxacin and as such, we decided to further perform quantitative assays only against Gram-negative bacteria.

An interesting result was the moderate activity of some norfloxacin-thiazolidinedione hybrids against the two *Candida* strains, with diameters between 8 and 19 mm. Although fluoroquinolones are not known for their antifungal activity, literature reports suggest at least one case of norfloxacin analogues that inhibit fungal strains, most likely by exhibiting chitin-synthase (CHS) inhibitory activity [11].

#### 2.2.2. Antimicrobial Activity—In Vitro Quantitative Assay

As a consequence of the initial antimicrobial screening, the quantitative determination of the antibacterial activity was determined only against Gram-negative bacteria. In order to better assess the antimicrobial activity, we decided to increase the number of Gram-negative bacterial strains to 5. As can be inferred from Table 2, the antimicrobial potential of the newly synthesized compounds significantly varied among each other. However, the minimal inhibitory concentration, of tested compounds, required to inhibit microbial growth was higher than that of norfloxacin (**1-Nor**).

When considering the value of MIC, the activity against the *E. coli* strains, compound **7a** showed the most promising results, followed by compound **2**. For *S. enteritidis* compound **2** and **7a** had the same apparent efficiency, while for *S. typhimurium* in addition to compounds **2** and **7a**, **7b** and **7f** presented similar values (Table 2). In case of the *P. aeruginosa* strain, the overall MIC values of the tested compounds including norfloxacin were high, with a potential good efficiency for compounds **2**, **7a** and **7b**. The MIC_50_ analysis results revealed similar observations as for MIC. In addition, for *S. enteritidis*, compound **7b** showed similar activity with **2** and **7a**, while for *P. aeruginosa*, the compound **7b** had a good overall efficiency comparable to compounds **2** and **7a**, similar with results norfloxacin.

From a structural point of view, it is no surprise that the highly reactive intermediate **2** has a good antibacterial activity. However, due to its chemical characteristics it is an unlikely drug candidate, and the data concerning its’ biological activity serves mainly for the development of structure-activity relationships within the series.

In the case of compound **6** (obtained by the alkylation reaction between the chloroacetamide intermediate **2** and thiazolidine-2,4-dione) we can observe a decrease of antibacterial activity against all strains tested. As a result, we can state that the addition of an unsubstituted thiazolidinedione moiety, indirectly linked to the C-7 piperazine residue of norfloxacin, is not beneficial for overall antibacterial activity.

**Figure 3:**
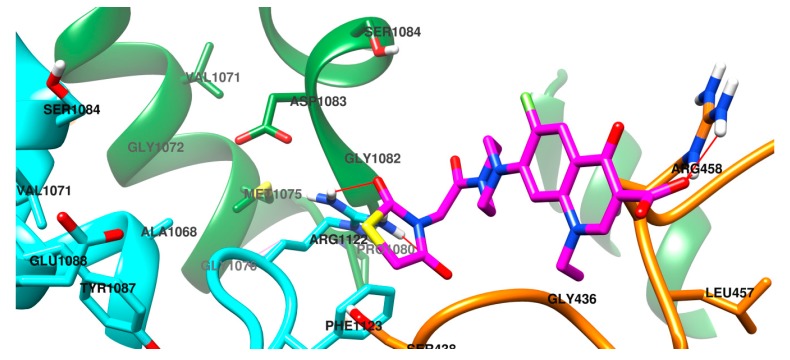
Molecule of compound **6** bound in the active site of bacterial DNA gyrase. The two subunits of gyrase are depicted in orange (GyrB), respectively green (GyrA). The cross-linking made by compound **6** between the two subunits is depicted with red lines.

The new norfloxacin–thiazolidinedione hybrids **7a**–**f**, that also possess an extra phenyl ring (Figure 3), are more potent compared with the unsubstituted compound **6**. This suggests that the extra ring is beneficial. Considering the presence or absence of further substitution on the phenyl ring, results suggest that no substitution is preferable as the most active compound seems to be **7a**. The most tolerated substituent appears to the *p*-methoxy (**7b**), that causes the maintenance of a similar antibacterial effect with the non-substituted molecules.

These results seem to correlate with those reported by other research endeavors that signalized the problematic outcome of the initiative of increasing C7 fluoroquinolone substituent’s size [6,12,13,14,21]. Improved biological activity can be obtain if a new molecular binding mode is achieved for the quinolone-topoisomerase-DNA ternary complex [3,9,11].

#### 2.2.3. Anti-Biofilm Activity Assay

Biofilm formation explains the ability of microorganisms to form surface-attached communities and thus offers pathogens the opportunity to adapt to various habitats, to better resist antibiotic action, to form synergic polymicrobial entities and as a consequence the increased pathogenicity with more severe infections, increased antibiotic resistance and increased mortality [22,27,32,33].

Compared with direct antimicrobial activity, anti-biofilm activity has the advantage that it decreases pathogenicity but is not accompanied by selection pressure and thus it does not cause the appearance of resistant strains [29].

Taking into account the fact that anti-biofilm activity usually has different biological mechanisms, and is most often independent from the direct antimicrobial effects, we decided to test all compounds using a broad spectrum of Gram-positive and Gram-negative bacteria, and also fungal strains (as seen in Table 3).

The tested compounds showed a very good anti-biofilm activity against *S. aureus,* with MBEC values up to 16 times smaller than the standard berberine. The reactive intermediate **2** and the unsubstituted thiazolidinedione derivative **6** proved to be especially active. Compounds that had an extra phenyl ring added to their C-7 substituent turned out to be less active (**7a**, **7b**, **7c**) if the nucleus did not contain an extra halogen substituent. The presence of extra fluorine or chlorine substituents also yielded very active molecules (**7d**, **7e** and **7f).** Multi-ringed molecules that specifically inhibit *S. aureus* biofilm usually do so by inhibiting sortase A, a key enzyme in Gram-positive bacteria virulence [34,35,36,37,38].

The anti-biofilm activity against most bacterial strains is varied and usually more reduced or at best similar with berberine. One of the most promising compounds is **6,** which has good activity against the two Gram-positive strains and also against *E. coli*.

The new norfloxacin-thiazolidinediones seem to have a modest effect on *Candida sp*. biofilm formation. Although similar research showed that thiazolidine-2,4-dione is present in several anti-biofilm agents [22,24] active against *Candida*, the hybridization with norfloxacin leads to a dramatic decrease in effect.

When analyzing anti-biofilm activity as a whole, it is apparent that most of the compounds are active against all strains at concentrations equal or smaller than that of the standard. As such, it is very likely that the compounds act via a non-specific mechanism in order to inhibit biofilm formation and microbial adhesion.

### 2.3. Molecular Docking Study

DNA gyrase plays a crucial role in interconversion of the DNA between the relaxed and the supercoiled forms, maintaining its normal topological state in the cell [6]. DNA gyrase is made up 2 GyrA and 2 GyrB subunits. GyrA is comprised of an N-terminal domain (GHKL), responsible for DNA G-segment cleavage, and a C-terminal domain topoisomerase-primase (TOPRIM) responsible for substrate recognition and DNA wrapping. The TOPRIM fragment has a Rossman fold structural motif, with a β-sheet comprised by two groups of α-helices [39].

GyrB is comprised of an N-terminal (WHD) domain important for ATPase binding and its hydrolysis, and a C-terminal domain (CTD) responsible with interactions with DNA and GyrA [6,11,14]. The absence of gyrases in the mammalian cells makes them a good target for development of novel antimicrobial drugs [6]. This advantage of druggability was validated by the approval and further widespread use of fluoroquinolones.

While fluoroquinolones have a DNA poisoning activity, other molecules, like aminocoumarins, inhibit DNA gyrase in a competitive manner by perturbing the ATPase activity. This takes place in the N-terminal domain of the GyrB subunit, which is responsible for the catalytic activity and has a pocket for ATPase, providing energy to the whole catalytic system, due to Mg^2+^-mediated ATP-hydrolysis. However, the successful clinical use of aminocoumarins is hindered by low activity against Gram-negative strains and poor pharmacokinetics [6,40].

As such, molecules that act similarly to fluoroquinolones, by poisoning the topoisomerases are still preferred. The results of the molecular docking study are presented in the Table 4. An increase in interaction with the bacterial topoisomerase is noted with increasing molecule substitution, starting with norfloxacin (**1**), passing through the intermediate **2**, the thiazolidine-2,4-dione derivative (**6**) and the **7a**–**f** derivatives, having in their structures a supplementary phenyl nucleus. This can be quantified by the evolution of binding energies from −6.35 kcal/mol in the case of norfloxacin (**1**), reaching −6.66 kcal/mol in case of compound **6** derived from thiazolidine-2,4-dione. Derivatization of compound **6** by the insertion of a phenyl ring, optionally substituted in the *para* position, leads to an increase in affinity to values ranging between −7.63 and −7.89 kcal/mol. Insertion of a chlorine atom in *ortho* position of compound **7e** led to compound **7f**, with a much better interaction with the bacterial topoisomerase (ΔG = −8.16 kcal/mol).

Conformations resulting from the molecular docking study were clustered in 2**** Å clusters. The final compounds **7a-f** are predicted to have a more chaotic binding pattern than the parent compound, norfloxacin. This became apparent because of the higher number of clusters than those for norfloxacin.

Figure 2 depicts the docked complex between norfloxacin and DNA gyrase. Norfloxacin interacts with the alcohol group from the side chain of Ser1084 via the carboxyl moiety, while another polar contact is made with Arg458, through the terminal secondary amine from the piperazine ring.

The predicted binding pose for compound **6** is depicted in Figure 3. The cross-linking between the two units of the bacterial gyrase is predicted to take place, but by interacting with a different amino acid residue. The oxygen from the thiazolidine-2,4-dione ring is predicted to interact with the sidechain of Arg1122 from GyrA, while the carboxyl and the carbonyl from the quinolone system is predicted to interact with Arg458 from GyrB. The sp3 carbon atom, which gives structural flexibility, allows the molecule to bend between the piperazine nucleus and thiazolidine-2,4-dione.

In the case of compounds **7a**–**f** an extra nucleus is attached via a CH bridge to the thiazolidine-2,4-dione ring. Docking studies predict that these compounds also maintain the similar interaction mode predicted for compound **6**, with key interactions with Arg1122 from GyrA, and Arg458 from GyrB. A major difference is represented by the fact that compounds **7a**–**f** have the ability to form a complex interaction with Arg1122, involving simultaneously both the oxygen from thiazolidine-2,4-dione ring, and also the oxygen from the acetamide bridge.

**Figure 4:**
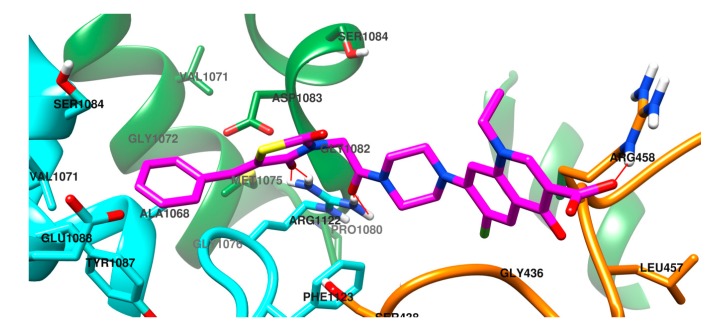
Molecule of compound **7a** bound in the active site of bacterial DNA gyrase. The two subunits of gyrase are depicted in orange (GyrB), respectively green (GyrA). The cross-linking made by compound **7a** between the two subunits is depicted with red lines.

Furthermore, due to the elongation of the molecular scaffold, the new molecules need to fold slightly in order to fit in the binding site. As a result, when docked, they adopt a v-shaped conformation made possible due to the structural flexibility provided by the methylene bridge, as shown in Figure 4**.** Due to this improved binding mode, seen for compounds **7a**–**f**, it is plausible that these derivatives are more efficient in binding to the target enzyme and thus could provide stronger antimicrobial activity than that seen for compound **6**.

Also, this new binding mode predicted for the norfloxacin-thiazolidinedione hybrids could suggest that they are capable of interacting with the topoisomerase-DNA complex in a manner that does not require the interaction with the Ser and Asp residues form GyrA. As a frequent reason of fluoroquinolone resistance is represented by mutations that cause the replacement of these amino acid residues from GyrA binding site [3], this suggest that the new molecules could be active against bacterial strains resistant to classic quinolones already used in therapy.

### 2.4. In Silico ADMET Study

Some chemical descriptors of the molecules **1**, **2**, **6** and **7a**–**f** are presented in Table 5. It is obvious that substitution of the parent compound with supplementary residues leads to the increase of the molecular weight, number or rotatable bonds, molecular surface, molecular polar surface. 

The substitution of norfloxacin with bulky residues, with or without lipophilic atoms as chlorine or fluorine, leads to increased molecular lipophilicity, expressed as logarithm of octanol:water partition, and the consequent decreases their water solubility.

Gastrointestinal absorption predicted using the pkCSM QSAR tool, shown in Table 6, has evaluated as theoretical absorption in Caco2 cells (human epithelial intestinal cells) expressed as score and as percent of a dose absorbed in the blood from the gastrointestinal tract. Norfloxacin (**1-Nor**) and its chloroacetamide derivative **2** are predicted to have high gastrointestinal absorption, both in Caco2 permeability score both (>1.2) and intestinal absorption (>83% both). Introducing in the norfloxacin molecule the thiazolidine-2,4-dione-acetamide fragment leads to drastically reduced absorption (Caco2 permeability = 0.984 and intestinal absorption = 52.33%). Supplementary substitution of compound **6** with an aromatic ring in the 5 position leads to a supplementary decreasing of Caco2 cells permeability for compounds **7a**–**f**, ranging between 0.722–0.841.

This result is interesting, especially in the case of the halogenated molecules, with a lipophilicity much higher than norfloxacin (**1-Nor**). Similarly, the predicted intestinal absorption is reduced compared to norfloxacin, independently of nature of the aromatic ring substitution, with values between 58.97–64.77%. These findings suggest that the gastrointestinal absorption depends on multiple molecular descriptors, lipophilicity being not sufficient to describe the potential absorption of a molecule [41].

The theoretical results of the ADMET study, shown in Table 6, indicate that norfloxacin (**1-Nor**) is a substrate of Pgp, an experimentally proven fact according to literature [42,43,44]. This property could be connected with the fact that fluoroquinolone are also substrates for numerous efflux systems present in some bacteria (e.g., *E. coli* genome has 29 potential efflux pumps). As a result of over-expression of these efflux pumps, efflux-based fluoroquinolone resistance can occur [3,10]. The capacity to efflux fluoroquinolones is coupled with the tendency to acquire additional fluoroquinolone resistance determinants.

The first thiazolidine-2,4-dione derivative in our series (compound **6**) is predicted to be a substrate of P-glycoprotein, as its parent compound. Insertion of an aromatic ring in the position 5 of the thiazolidinedione nucleus in compound **6** results in the disappearance of the susceptibility of compounds **7a**–**f** of being a substrate P-glycoprotein. As such, this may suggest that the new hybrids have a smaller chance of triggering efflux-based fluoroquinolone resistance.

None of the compounds in our series are predicted to be blood-brain barrier permeants. None of the final compounds **6** and **7a**–**f** are predicted to be inhibitors of CYP1A2, CYP2C19 or CYP2D6, but all are predicted to be inhibitors of CYP2C9. Reduce CYP interaction usually translates in less significant changes of metabolism interactions with other drug molecules and also reduced hepatic toxicity.

## 3. Materials and Methods

### 3.1. General Information

Chemicals used for the synthesis, isolation and purification were purchased from Merck (Darmstadt, Germany), Sigma-Aldrich (Taufkirchen, Germany) and Alfa Aesar (Karlsruhe, Germany). All chemicals were of analytical reagent grade purity. Thin-layer chromatography was performed on silica gel sheets, with UV-light visualization, for primary assessment of compounds’ purity. This initial data was later confirmed with supplementary investigations using HPLC analyses (Supplementary Materials). The melting points are uncorrected and were obtained by using an MPM-H1 melting point apparatus (Schorpp Gerätetechnik, Überlingen, Germany). IR spectra were recorded on a FT/IR 6100 spectrometer (Jasco, Cremella, Italy) after compression in anhydrous KBr pellets under vacuum. Water and carbon dioxide signals were removed from the recorded IR spectrum using the computer interface software Spectra Manager and assignment of signals was assisted by Know It All 7.8 by Bio-Rad Laboratories (Hercules, CA, USA).

MS spectra were obtained by using an Agilent 1100 series, in positive ionization mode (electrospray ionization ESI) with an Agilent Ion Trap SL mass spectrometer instrument (Agilent Technologies, Santa Clara, CA, USA). The ^1^H-NMR and ^13^C-NMR were recorded on an Avance NMR spectrometer (Bruker, Karlsruhe, Germany) operating at 500 MHz and 125 MHz, respectively, in DMSO-d_6_ as solvent. Chemical shift values are reported in δ units, relative to TMS as internal standard. All spectral data were in accordance with the proposed chemical structures.

### 3.2. Chemistry

7-(4-(2-Chloroacetyl)piperazin-1-yl)-1-ethyl-6-fluoro-4-oxo-1,4-dihydroquinoline-3-carboxylic acid (**2**) was already reported in the literature and was re-synthesized by our group using the previously reported protocol [45,46]. Light yellow-brown solid; mp = 257–258 °C (lit. 261–263 °C [46], 241–242 °C [45]); yield = 39%; FT IR (KBr) ν_max_ cm^−1^: 1728 (C=O acid str), 1715 (C=O amide str), 1704 (C=O ketone str); MS: *m*/*z* = 396.3 (M+1, with isotopically pattern due to ^35^Cl and ^37^Cl); ^1^H-NMR δ: 1.43 (t, *J*_H-H_ = 7 Hz, 3H, -CH_3_), 3.57 (broad, 4H, piperazine), 3.69 (broad, 4H, piperazine), 4.46 (s, 2H, -CO-CH_2_-), 4.62 (q, *J*_H-H_ = 7 Hz, 2H, -CH_2_-CH_3_), 7.23 (d, *J*_H-F_ = 7.5 Hz, 1H, H-8 of quinolone), 7.95 (d, *J*_H-F_ = 13 Hz, 1H, H-5 of quinolone), 8.97 (s, 1H, H-2 of quinolone); ^13^C-NMR δ: 14.87 (CH_3_-), 36.73 (-CH_2_-), 41.25 (-CH_2_-), 43.08 (-CH_2_-), 43.35 (-CH_2_-), 49.60 (-CH_2_-), 51.19 (-CH_2_-), 106.81, 107.63 (C-3 of quinolone), 111.40 (*J*_C-F_ = 23.75 Hz, C-5 of quinolone), 120.47, 137.65, 144.87, 149.12 (C-2 of quinolone), 153.29 (*J*_C-F_ = 23.75 Hz, C-6 of quinolone), 165.32 (C=O), 172.06 (C=O), 175.84 (C=O).

The procedure applied for the synthesis of the intermediate compounds **5a**–**e** was performed using a previously reported protocol [47] and the structural data were consistent with the previously reported characterizations [47,48,49].

### 3.3. General Procedure for the Synthesis of the Final Compounds 6, 7a–e

In a round bottom glass flask, 0.396 g (1 mmol) of intermediate compound **2**, 0.414 g (3 mmol) of anhydrous K_2_CO_3_ and 0.166 g (1 mmol) of anhydrous KI were poured over 75 mL of anhydrous acetone. The mixture was refluxed on a water bath for 3 h. Once the completion of the reaction was confirmed by TLC, the acetone was removed using a rotational evaporator. The resulted solid was mixed with ice cold water and sodium chloride, while a 10% sulfuric acid solution was added dropwise in order to obtain the maximum amount of precipitate of the desired final product. The flask was shaken from time to time, after that the separated solid was filtered under vacuum and washed with fresh water. After drying, the resulted solid was crystallized twice from acetone, in order to get the pure final products.

*7-(4-(2-(2,4-Dioxothiazolidin-3-yl)acetyl)piperazin-1-yl)-1-ethyl-6-fluoro-4-oxo-1,4-dihydroquinoline-3-carboxylic acid* (**6**): white solid; carbonization over 275 °C; yield = 63%; FT IR (KBr) ν_max_ cm^−1^: 1760 (C=O TZD str), 1731 (C=O acid str), 1713 (C=O amide str), 1698 (C=O ketone str), 1679 (C=O TZD str); MS: *m*/*z* = 477.6 (M+1); ^1^H-NMR δ: 1.43 (t, *J*_H-H_ = 7 Hz, 3H, -CH_3_), 3.41 (broad, 4H, piperazine), 3.68 (broad, 2H, piperazine), 3.76 (broad, 2H, piperazine), 4.34 (s, 2H, -CO-CH_2_-), 4.52 (s, 2H, C5-TZD), 4.61 (q, *J*_H-H_ = 7 Hz, 2H, -CH_2_-CH_3_), 7.24 (d, *J*_H-F_ = 7.5 Hz, 1H, H-8 of quinolone), 7.97 (d, *J*_H-F_ = 13 Hz, 1H, H-5 of quinolone), 8.97 (s, 1H, H-2 of quinolone); ^13^C-NMR δ: 14.87 (-CH_2_-), 34.40 (C5-TZD), 41.83 (-CH_2_-), 42.32 (-CH_2_-), 42.79 (-CH_2_-), 44.32 (-CH_2_-), 49.59 (-CH_2_-), 50.05 (-CH_2_-), 106.92, 107.63 (C-3 of quinolone), 111.74 (*J*_C-F_ = 23.75 Hz, C-5 of quinolone), 120.08, 137.63, 145.62, 149.11 (C-2 of quinolone), 153.35 (*J*_C-F_ = 23.75 Hz, C-6 of quinolone), 163.87 (C=O), 166.55 (C=O), 172.11 (C=O), 172.43 (C=O), 176.67 (C=O).

*(Z)-7-(4-(2-(5-Benzylidene-2,4-dioxothiazolidin-3-yl)acetyl)piperazin-1-yl)-1-ethyl-6-fluoro-4-oxo-1,4-dihydroquinoline-3-carboxylic acid* (**7a**): white solid; mp = 240 °C; yield = 58%; FT IR (KBr) ν_max_ cm^−1^: 1755 (C=O TZD str), 1725 (C=O acid str), 1715 (C=O amide str), 1698 (C=O ketone str), 1684 (C=O TZD str); MS: *m*/*z* = 565.2 (M+1); ^1^H-NMR δ: 1.43 (t, *J*_H-H_ = 7 Hz, 3H, -CH_3_), 3.54 (broad, 4H, piperazine), 3.70 (broad, 2H, piperazine), 3.80 (broad, 2H, piperazine), 4.62 (q, *J*_H-H_ = 7 Hz, 2H, -CH_2_-CH_3_), 4.72 (s, 2H, -CO-CH_2_-), 7.21 (d, *J*_H-F_ = 7.5 Hz, 1H, H-8 of quinolone), 7.27 (t, *J*_H-H_ = 7.5 Hz, 1H, Ar), 7.42 (m, 2H, Ar), 7.67 (d, *J*_H-H_ = 7.5 Hz, 2H, Ar), 7.96 (d, *J*_H-F_ = 13 Hz, 1H, H-5 of quinolone), 7.99 (s, 1H, -CH=), 8.98 (s, 1H, H-2 of quinolone); ^13^C-NMR δ: 14.87 (-CH_2_-), 42.34 (-CH_2_-), 42.80 (-CH_2_-), 43.06 (-CH_2_-), 43.82 (-CH_2_-), 49.60 (-CH_2_-), 50.35 (-CH_2_-), 107.04, 107.62 (C-3 of quinolone), 111.70 (*J*_C-F_ = 22.75 Hz, C-5 of quinolone), 120.05, 122.17, 123.26, 130.88, 132.17, 133.50, 137.68, 145.84, 149.14 (C-2 of quinolone), 153.30 (*J*_C-F_ = 247 Hz, C-7 of quinolone), 163.48 (C=O), 165.78 (C=O), 167.94 (C=O), 168.43 (C=O), 176.75 (C=O).

*(Z)-1-Ethyl-6-fluoro-7-(4-(2-(5-(4-methoxybenzylidene)-2,4-dioxothiazolidin-3-yl)acetyl)piperazin-1-yl)-4-oxo-1,4-dihydroquinoline-3-carboxylic acid* (**7b**): yellow-white solid; mp = 205 °C; yield = 41%; FT IR (KBr) ν_max_ cm^−1^: 1760 (C=O TZD str), 1730 (C=O acid str), 1716 (C=O amide str), 1704 (C=O ketone str), 1690 (C=O TZD str), 1258 (aryl-alkyl ether asymm str), 1090 (aryl-alkyl ether symm str); MS: *m*/*z* = 595.3 (M+1); ^1^H-NMR δ: 1.44 (t, *J*_H-H_ = 7 Hz, 3H, -CH_3_), 3.44 (broad, 4H, piperazine), 3.70 (broad, 2H, piperazine), 3.80 (broad, 2H, piperazine), 3.85 (s, 3H, -OCH_3_), 4.61 (q, *J*_H-H_ = 7 Hz, 2H, -CH_2_-CH_3_), 4.70 (s, 2H, -CO-CH_2_-), 7.15 (d, *J*_H-H_ = 8.5 Hz, 2H, Ar), 7.25 (d, *J*_H-F_ = 7.5 Hz, 1H, H-8 of quinolone), 7.63 (d, *J*_H-H_ = 8.5 Hz, 2H, Ar), 7.94 (m, 2H, -CH= and H-5 of quinolone), 8.97 (s, 1H, H-2 of quinolone); ^13^C-NMR δ: 14.87 (-CH_2_-), 40.89 (-CH_2_-), 42.09 (-CH_2_-), 43.10 (-CH_2_-), 44.38 (-CH_2_-), 49.70 (-CH_2_-), 50.09 (-CH_2_-), 55.96 (-CH_3_), 105.42, 107.64 (C-3 of quinolone), 111.68 (*J*_C-F_ = 22.75 Hz, C-5 of quinolone), 115.35, 120.17, 126.29, 131.30, 131.40, 132.40, 137.66, 144.22, 149.11 (C-2 of quinolone), 153.34 (*J*_C-F_ = 247 Hz, C-6 of quinolone), 161.25 (ArC-O), 164.30 (C=O), 166.56 (C=O), 170.09 (C=O), 173.04 (C=O), 177.31 (C=O).

*(Z)-7-(4-(2-(5-(4-(Dimethylamino)benzylidene)-2,4-dioxothiazolidin-3-yl)acetyl)piperazin-1-yl)-1-ethyl-6-fluoro-4-oxo-1,4-dihydroquinoline-3-carboxylic acid* (**7c**): orange solid; carbonization over 265 °C; yield = 43%; FT IR (KBr) ν_max_ cm^−1^: 1754 (C=O TZD str), 1730 (C=O acid str), 1713 (C=O amide str), 1696 (C=O ketone str), 1678 (C=O TZD str); MS: *m*/*z* = 608.4 (M+1); ^1^H-NMR δ: 1.44 (t, *J*_H-H_ = 7 Hz, 3H, -CH_3_), 3.04 (s, 6H, N(CH_3_)_2_), 3.44 (broad, 4H, piperazine), 3.69 (broad, 2H, piperazine), 3.79 (broad, 2H, piperazine), 4.60 (q, *J*_H-H_ = 7 Hz, 2H, -CH_2_-CH_3_), 4.67 (s, 2H, -CO-CH_2_-), 6.73 (d, *J*_H-H_ = 9 Hz, 2H, Ar), 7.25 (d, *J*_H-F_ = 7.5 Hz, 1H, H-8 of quinolone), 7.50 (d, *J*_H-H_ = 9 Hz, 2H, Ar), 7.83 (s, 1H, -CH=), 7.96 (d, *J*_H-F_ = 13 Hz, 1H, H-5 of quinolone), 8.97 (s, 1H, H-2 of quinolone); ^13^C-NMR δ: 14.87 (-CH_2_-), 40.80 (-CH_3_), 41.49 (-CH_2_-), 42.60 (-CH_2_-), 44.21 (-CH_2_-), 45.71 (-CH_2_-), 49.69 (-CH_2_-), 50.21 (-CH_2_-), 106.76, 107.63 (C-3 of quinolone), 111.43 (*J*_C-F_ = 22.75 Hz, C-5 of quinolone), 112.53, 113.12, 120.31, 120.86, 131.89, 132.34, 137.70, 144.71, 149.10 (C-2 of quinolone), 151.70, 153.33 (*J*_C-F_ = 247 Hz C-6 of quinolone), 165.35 (C=O), 165.81 (C=O), 167.44 (C=O), 174.59 (C=O), 176.89 (C=O).

*(Z)-1-Ethyl-6-fluoro-7-(4-(2-(5-(4-fluorobenzylidene)-2,4-dioxothiazolidin-3-yl)acetyl)piperazin-1-yl)-4-oxo-1,4-dihydroquinoline-3-carboxylic acid* (**7d**): white solid; carbonization over 280 °C; yield = 68%; FT IR (KBr) ν_max_ cm^−1^: 1755 (C=O TZD str), 1735 (C=O acid str), 1714 (C=O amide str), 1700 (C=O ketone str), 1685 (C=O TZD str); MS: *m*/*z* = 583.3 (M+1); ^1^H-NMR δ: 1.44 (t, *J*_H-H_ = 7 Hz, 3H, -CH_3_), 3.44 (broad, 4H, piperazine), 3.69 (broad, 2H, piperazine), 3.79 (broad, 2H, piperazine), 4.60 (q, *J*_H-H_ = 7 Hz, 2H, -CH_2_-CH_3_), 4.71 (s, 2H, -CO-CH_2_-), 7.23 (d, *J*_H-F_ = 7.5 Hz, 1H, H-8 of quinolone), 7.42 (d, *J*_H-H_ = 9 Hz, 2H, Ar), 7.73 (d, *J*_H-H_ = 9 Hz, 2H, Ar), 7.93 (d, *J*_H-F_ = 13 Hz, 1H, H-5 of quinolone), 7.99 (s, 1H, -CH=), 8.96 (s, 1H, H-2 of quinolone); ^13^C-NMR δ: 14.87 (-CH_2_-), 41.90 (-CH_2_-), 43.20 (-CH_2_-), 49.60 (-CH_2_-), 50.07 (-CH_2_-), 106.79, 107.62 (C-3 of quinolone), 111.66 (*J*_C-F_ = 22.75 Hz, C-5 of quinolone), 117.50 (*J*_C-F_ = 21 Hz), 120.09, 126.21, 126.91, 130.01, 135.19, 136.69, 149.10 (C-2 of quinolone), 144.99, 153.30 (*J*_C-F_ = 247 Hz C-6 of quinolone), 163.57 (*J*_C-F_ = 249 Hz), 163.80 (C=O), 165.74 (C=O), 166.55 (C=O), 167.45 (C=O), 176.65 (C=O).

*(Z)-7-(4-(2-(5-(4-Chlorobenzylidene)-2,4-dioxothiazolidin-3-yl)acetyl)piperazin-1-yl)-1-ethyl-6-fluoro-4-oxo-1,4-dihydroquinoline-3-carboxylic acid* (**7e**): gray solid; carbonization over 270 °C; yield = 57%; FT IR (KBr) ν_max_ cm^−1^: 1758 (C=O TZD str), 1729 (C=O acid str), 1710 (C=O amide str), 1698 (C=O ketone str), 1688 (C=O TZD str); MS: *m*/*z* = 599.2 (M+1); ^1^H-NMR δ: 1.44 (t, *J*_H-H_ = 7 Hz, 3H, -CH3), 3.44 (broad, 4H, piperazine), 3.70 (broad, 2H, piperazine), 3.79 (broad, 2H, piperazine), 4.61 (q, *J*_H-H_ = 6.5 Hz, 2H, -CH_2_-CH_3_), 4.72 (s, 2H, -CO-CH_2_-), 7.25 (d, *J*_H-F_ = 7.5 Hz, 1H, H-8 of quinolone), 7.64 (d, *J*_H-H_ = 8.5 Hz, 2H, Ar), 7.68 (d, *J*_H-H_ = 8.5 Hz, 2H, Ar), 7.95 (d, *J*_H-F_ = 13 Hz, 1H, H-5 of quinolone), 7.99 (s, 1H, -CH=), 8.97 (s, 1H, H-2 of quinolone); ^13^C-NMR δ: 14.87 (-CH_2_-), 41.90 (-CH_2_-), 43.23 (-CH_2_-), 44.01 (-CH_2_-), 49.60 (-CH_2_-), 50.03 (-CH_2_-), 106.88, 107.64 (C-3 of quinolone), 111.75 (*J*_C-F_ = 22.75 Hz, C-5 of quinolone), 120.12, 122.01, 122.65, 129.98, 132.35, 132.71, 135.90, 137.63, 145.64, 149.11 (C-2 of quinolone), 153.36 (*J*_C-F_ = 247 Hz C-6 of quinolone), 163.77 (C=O), 165.77 (C=O), 166.55 (C=O), 167.32 (C=O), 176.67 (C=O).

*(Z)-7-(4-(2-(5-(2,4-Dichlorobenzylidene)-2,4-dioxothiazolidin-3-yl)acetyl)piperazin-1-yl)-1-ethyl-6-fluoro-4-oxo-1,4-dihydroquinoline-3-carboxylic acid* (**7f**): white solid; carbonization over 270 °C; yield = 63%; FT IR (KBr) ν_max_ cm^−1^: 1754 (C=O TZD str), 1726 (C=O acid str), 1711 (C=O amide str), 1701 (C=O ketone str), 1684 (C=O TZD str); MS: *m*/*z* = 634.2 (M+1); ^1^H-NMR δ: 1.43 (t, *J*_H-H_ = 7 Hz, 3H, -CH_3_), 3.58 (broad, 4H, piperazine), 3.70 (broad, 2H, piperazine), 3.75 (broad, 2H, piperazine), 4.12 (s, 2H, -CO-CH_2_-), 4.61 (m, 2H, -CH_2_-CH_3_), 7.22 (d, *J*_H-F_ = 7.5 Hz, 1H, H-8 of quinolone), 7.40 (d, *J*_H-H_ = 8 Hz, 1H, Ar), 7.68–7.70 (m, 2H, Ar), 7.86 (s, 1H, -CH=), 7.99 (d, *J*_H-F_ = 13 Hz, 1H, H-5 of quinolone), 8.98 (s, 1H, H-2 of quinolone); ^13^C-NMR δ: 14.87 (-CH_2_-), 41.69 (-CH_2_-), 42.53 (-CH_2_-), 46.76 (-CH_2_-), 49.58 (-CH_2_-), 49.69 (-CH_2_-), 50.23 (-CH_2_-), 106.97, 107.66 (C-3 of quinolone), 111.69 (*J*_C-F_ = 22.75 Hz, C-5 of quinolone), 125.99, 128.51, 128.83, 130.40, 130.49, 130.62, 135.83, 135.91, 137.70, 146.34, 149.09 (C-2 of quinolone), 153.54 (*J*_C-F_ = 247 Hz C-6 of quinolone), 167.45 (C=O), 167.90 (C=O), 172.25 (C=O), 174.49 (C=O), 176.91 (C=O).

### 3.4. Biological Assays

The strains used for the evaluation were reference strains and their identity was reconfirmed using the VITEK 1 automatic system.

#### 3.4.1. Antimicrobial Activity—Initial In Vitro Qualitative Screening Study

The purpose of the initial screening evaluation was to select the derivatives with increased antimicrobial potential on Gram-positive, Gram-negative or yeast strains. A total of nine standard microbial strains were used—four Gram-positive strains: *Staphylococcus aureus* ATCC 6538P, *Enterococcus faecalis* ATCC 29212, *Listeria monocytogenes* ATCC 13932 and *Bacillus cereus* ATCC 11778; three Gram-negative: *Escherichia coli* ATCC 10536, *Escherichia coli* ATCC 25922 and *Salmonella enteritidis* ATCC 13076; and two Candida strains: *Candida albicans* ATCC 90028 and *Candida parapsilosis* ATCC 22019. Standard antimicrobials and antifungals were used as controls: norfloxacin for bacteria and ketoconazole for fungi. The screening step was performed using an adapted disk diffusion test, following EUCAST standards [22,50]. The newly synthesized compounds and the standards (norfloxacin and ketoconazole) were suspended in sterile dimethyl sulfoxide (DMSO) to a concentration of 2.327 mg × mL^−1^. Microbial colonies (24 h old) grown on Mueller-Hinton agar were used to prepare 1 mL suspension adjusted at 0.5 McFarland density in saline. This was used to flood 9 cm diameter Petri dishes with Mueller-Hinton agar. The excess fluid was removed and sterile filter paper discs of 5 mm diameter were placed in a radial model. A total amount of 10 µL was placed on each filter paper disk and the plates were allowed to incubate for 18 h at 35 ± 2 °C and 48 h at 28 °C for the fungal strains [22]. Antimicrobial activity was assessed as the diameter of the growth inhibition area, measured in mm.

#### 3.4.2. Antimicrobial Activity—In Vitro Quantitative Assay

The quantitative evaluation included the MIC method for five Gram negative standard strains were used: *Escherichia coli* ATCC 10536, *Escherichia coli* ATCC 25922, *Salmonella enteritidis* ATCC 13076, *Salmonella typhimurium* ATCC 14028 and *Pseudomonas aeruginosa* ATCC 27853.

The quantitative evaluation was performed according to the modified EUCAST protocols [22,50]. The evaluation was performed using 96-wells microplates containing liquid Mueller-Hinton medium inoculated with 20 µL from the microbial inoculum. The stock solutions of the tested compounds were prepared at concentrations of 2.327 mg × mL^−1^ in sterile DMSO. They were applied as two-fold serial dilutions ranging from 64 µg × mL^−1^ to 0.0625 µg × mL^−1^. The two-fold serial dilutions for norfloxacin were performed in triplicate and ranged from 16 µg × mL^−1^to 0.0156 µg × mL^−1^. The total broth volume was adjusted to 200 µg × mL^−1^. Norfloxacin (compound **1**) was used as standard antimicrobial agent. Positive culture controls and blank DMSO dilutions were also prepared. The plates were incubated for 24 h at 37 °C. The minimum inhibitory concentration (MIC) values were determined as the lowest concentration of the investigated compound that inhibited the growth of the microbial cultures (with the same optical density as the negative control), compared to the positive control, as established by a decreased value of absorbance at 450 nm (HiPo MPP-96, Biosan, Riga, Latvia). The MIC_50_ represents the MIC value at which ≥50% of the bacterial cells were inhibited in their growth, determined as the well with the closest OD to the average between the positive and negative control OD.

#### 3.4.3. Anti-Biofilm Activity Assay

The anti-biofilm activity was evaluated against a varied array of Gram-positive strains (*Staphylococcus aureus* ATCC 25923, *Enterococcus* *faecium* DSM 13950), Gram-negative strains (*Escherichia coli* ATCC 25922, *Pseudomonas aeruginosa* ATCC 27853) and fungi (*Candida albicans* ATCC 10231 and *Candida parapsilosis* ATCC 22019). We further tested the ability of the newly synthesized compounds to influence the biofilm development on inert substrata (plastic microplate wells) using the microtiter plate method. The stock solutions of the tested compounds were prepared at concentrations of 5 mg × mL^−1^ in sterile DMSO. Microbial suspensions with turbidity corresponding to McFarland 0.5 were cultivated in presence of binary concentration of the tested, ranging from 2500 µg × mL^−1^ to 5 µg × mL^−1^ and incubated at 37 °C for 24 h. The obtained cultures were discarded and the wells of the plates three times washed gently with sterile saline in order to remove the non-adherent microbial cells. Microbial biofilms developed on the plastic wells were fixed with ethanol for 5 min, stained with 1% violet crystal for 15 min, washed again with tap water and suspended in 33% acetic acid. The color intensity of the suspensions, correlated with the number of the microbial cells adherent to the inert substratum was assessed spectrophotometrically at 490 nm using a spectrophotometer (EZ Read 400, Biochrom, Cambridge, UK). All assays were done in triplicate [22,34,36].

### 3.5. Molecular Docking Study

The synthesized norfloxacin derivatives were docked into the active site of the DNA gyrase isolated from *Escherichia coli* (PDB code: 2XCT). The bioinformatic tool used in performing the molecular docking study was AutoDock 4.2 [51,52]. The present molecular docking study aimed on evaluation of the binding affinity of the compounds to the bacterial enzyme. The search space was cubic, with sides equal to 28 points, with 0.375 Å grid spacing. The Cartesian coordinates of the center of the search space were set to x = −9.883, y = 38.313 and z = 69.621. The choice of spatial parameters for the molecular docking study was performed so that the search center was positioned on the ciprofloxacin molecule with which the enzyme was initial co-crystallized. 200 conformations were searched for each compound. Visualization of the docking results was performed using UCSF Chimera [53].

### 3.6. In silico ADMET study

The compounds from the present paper were subjected to a theoretical ADMET study using Swiss-ADME [54] and pkCSM [55]. The ADMET study focused on the evaluation of molecular properties such as: polarity, lipophilicity, water solubility, gastrointestinal absorption, blood-brain barrier permeability, P-glycoprotein substrate potential and CYP450 inhibition potential.

To analyze the results of the ADMET study, a set of consecrated rules (Lipinski [56], Veber [57], Muegge [58] and Egan [59]) have been applied to assess the overall pharmacokinetic characteristics of our compounds.

## 4. Conclusions

A series of seven new norfloxacin-thiazolidinedione hybrids were synthesized with the aim of obtaining new fluoroquinolone hybrids that could be active even against resistant bacteria, while also having the ability to reduce pathogenicity by interfering with biofilm formation. All new molecular structures were confirmed using physicochemical and spectral data. The initial qualitative antimicrobial activity screening performed revealed that the new compounds are similar with norfloxacin in the fact that they are more active against Gram-negative strains. Quantitative assessment of the antibacterial activity showed that, overall, they have a diminished direct antibacterial activity compared with norfloxacin. However, unlike norfloxacin, the new derivatives have various degrees of anti-biofilm activity, more noticeable against *S. aureus*. The docking studies suggest that the new norfloxacin-thiazolidinedione hybrids have the ability to bind to the DNA gyrase in completely different manner, thus unlike classic fluoroquinolones they do not interact with the serine and aspartic acid residues that are often the subject to mutations leading to fluoroquinolones resistance. In silico ADMET studies predict that the new derivatives may also have the advantage that they are not substrates for Pgp.

## Figures and Tables

**Table 1:** The antimicrobial activity of the tested compounds (diameters of the microbial growth inhibition zone in mm).

	Antimicrobial Activity							Antifungal Activity	
	Gram-Positive				Gram-Negative				
Compound	*S. aureus*	*L. monocytogenes*	*B. cereus*	*E. faecalis*	*E. coli*	*E. coli*	*S. enteritidis*	*C. albicans*	*C. parapsilosis*
	ATCC 6538P	ATCC 13932	ATCC 11778	ATCC 29212	ATCC 10536	ATCC 25922	ATCC 13076	ATCC 90028	ATCC 22019
**1-Nor**	37	28	28	27	31	26	38	-	-
**2**	23	17	19	17	26	23	29	0	0
**6**	22	17	16	16	23	18	19	8	0
**7a**	20	0	17	16	29	26	32	9	0
**7b**	15	0	14	13	24	23	25	19	9
**7c**	11	0	10	0	23	21	19	0	0
**7d**	10	0	8	0	15	15	16	8	9
**7e**	16	0	11	0	17	14	0	11	0
**7f**	14	0	11	12	22	23	22	19	10
**Ketoconazole**	-	-	-	-	-	-	-	25	20
**DMSO**	0	0	0	0	0	0	0	0	0

“-“= not tested. Compounds and standards were applied as 10 µL solutions of 2.327 mg × mL^−1^ concentration.

**Table 2:** The minimum inhibitory concentrations MIC, MIC_50_(μg × mL^−1^) values for norfloxacin and the new norfloxacin–thiazolidinedione molecular hybrids.

Compound	Gram-Negative Bacteria									
	*E. coli* ATCC 25922		*E. coli* ATCC 10536		*S. typhimurium* ATCC 14028		*S. enteritidis* ATCC 13076		*P. aeruginosa* ATCC 27853	
	MIC	MIC_50_	MIC	MIC_50_	MIC	MIC_50_	MIC	MIC_50_	MIC	MIC_50_
**1-Nor**	0.0625	0.0312	0.125	0.0625	0.125	0.0625	0.0625	0.0312	1	0.5
**2**	0.5	0.125	1	0.25	2	1	1	0.5	2	1
**6**	2	1	8	2	32	8	8	4	16	8
**7a**	0.5	0.125	0.5	0.25	2	1	1	0.5	2	1
**7b**	0.25	0.125	4	1	2	0.5	2	0.5	2	0.5
**7c**	2	0.5	4	0.5	8	4	4	2	16	8
**7d**	1	0.25	4	1	8	2	8	4	128	64
**7e**	2	0.5	2	0.5	16	4	8	2	128	32
**7f**	1	0.5	2	0.5	2	1	2	1	8	2

**Table 3:** The minimal biofilm eradication concentration MBEC (μg × mL^−1^) values of the new norfloxacin–thiazolidinedione molecular hybrids.

Compound	Antimicrobial Activity				Antifungal Activity	
	Gram-Positive		Gram-Negative			
	*S. aureus*	*E. faecium*	*E. coli*	*P. aeruginosa*	*C. albicans*	*C. parapsilosis*
	ATCC 25923	DSM 13950	ATCC 25922	ATCC 27853	ATCC 10231	ATCC 22019
**1-Nor**	4.9	156.2	156.2	4.9	312.5	2500
**2**	4.9	78.1	78.1	78.1	312.5	312.5
**6**	4.9	9.8	39.0	312.5	312.5	2500.0
**7a**	19.5	625.0	156.2	625.0	156.2	156.2
**7b**	19.5	78.1	156.2	78.1	312.5	78.1
**7c**	39.0	156.2	156.2	78.1	312.5	312.5
**7d**	4.9	156.2	39.0	625.0	312.5	312.5
**7e**	9.8	78.1	78.1	312.5	312.5	312.5
**7f**	4.9	625.0	156.2	78.1	312.5	312.5
**Berberine**	78.1	156.2	625.0	625.0	312.5	312.5

**Table 4:** Predicted binding affinity and inhibition constants for the new norfloxacin–thiazolidinedione molecular hybrids with the topoisomerase-DNA complex.

Compound	Binding Affinity (kcal/mol)	Inhibition Constant (µM)	Cluster with the Best Conformation		Clusters	
			Average Binding Affinity (kcal/mol)	Number of Conformations	With Multiple Conformations	Total
**1-Nor**	−6.35	22.15	−6.20	6	7	7
**2**	−6.16	30.53	−5.76	18	4	10
**6**	−6.66	13.13	−5.91	13	7	17
**7a**	−7.82	1.85	−7.59	18	6	21
**7b**	−7.78	1.98	−7.41	6	6	24
**7c**	−7.71	2.23	−7.42	6	5	27
**7d**	−7.89	1.65	−6.98	13	6	29
**7e**	−7.63	2.55	−7.54	15	6	24
**7f**	−8.16	1.04	−7.91	9	8	25

**Table 5:** Physicochemical properties, lipophilicity and water solubility of the new derivatives.

Compound	MW	RB	HBA	HBD	Surface		LogP	Water Solubility	
					Total	Polar		mg/mL	Class
**1-Nor**	319	3	5	2	131.68	74.57	1.04	16.2	Very
**2**	395	5	5	1	159.09	82.85	1.28	0.168	Soluble
**6**	476	6	7	1	191.08	145.53	0.05	0.191	Soluble
**7a**	564	7	7	1	231.81	145.53	1.21	0.003	Moderately
**7b**	594	8	8	1	243.29	154.76	0.91	0.002	Moderately
**7c**	607	8	7	1	250.30	148.77	1.11	0.002	Moderately
**7d**	582	7	8	1	235.98	145.53	1.58	0.002	Moderately
**7e**	599	7	7	1	242.12	145.53	1.68	0.001	Moderately
**7f**	633	7	7	1	252.42	145.53	2.14	<0.001	Poorly

MW = molecular weight; RB = rotatable bonds; HBA = hydrogen bond acceptors; HBD = hydrogen bond donors.

**Table 6:** Predicted pharmacokinetics of the new compounds.

Compound	Absorption			BBBP	PgpS	CYP Inhibition			
	Caco2 Permeability	Intestinal Absorption	Class			1A2	2C19	2C9	2D6
**1-Nor**	1.254	83.73	High	No	Yes	No	No	No	No
**2**	1.270	96.52	High	No	No	No	No	No	No
**6**	0.984	52.33	Low	No	Yes	No	No	Yes	No
**7a**	0.823	61.39	Low	No	No	No	No	Yes	No
**7b**	0.747	62.85	Low	No	No	No	No	Yes	No
**7c**	0.841	58.97	Low	No	No	No	No	Yes	No
**7d**	0.774	64.77	Low	No	No	No	No	Yes	No
**7e**	0.727	62.56	Low	No	No	No	No	Yes	No
**7f**	0.722	63.74	Low	No	No	No	No	Yes	No

BBBP = blood brain barrier permeant; PgpS = P-glycoprotein substrate.

## Supplementary Materials

Chromatograms after HPLC analysis of the compounds are reported.
