# Supplementary material for: Design, Synthesis and Biological Evaluation of New Piperazin-4-yl-(acetyl-thiazolidine-2,4-dione) Norfloxacin Analogues as Antimicrobial Agents
Source: Molecules. 2019 Oct 31;24(21):3959. doi: 10.3390/molecules24213959 (PMC6864599; doi:10.3390/molecules24213959)

Chromatograms after HPLC analysis of the compounds:

Compound 2:

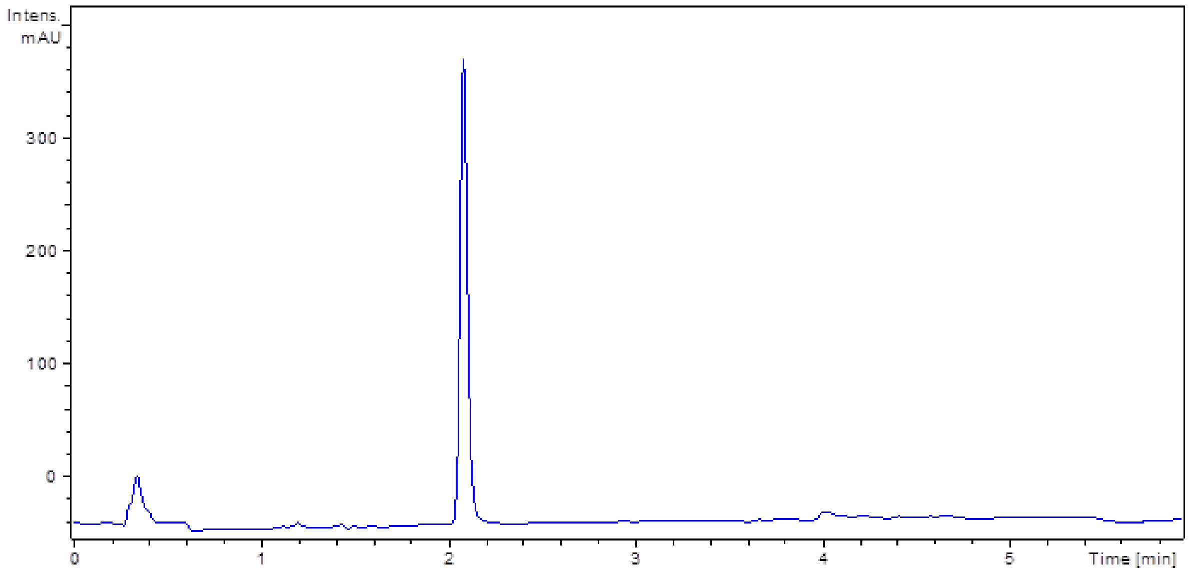

Compound 6:

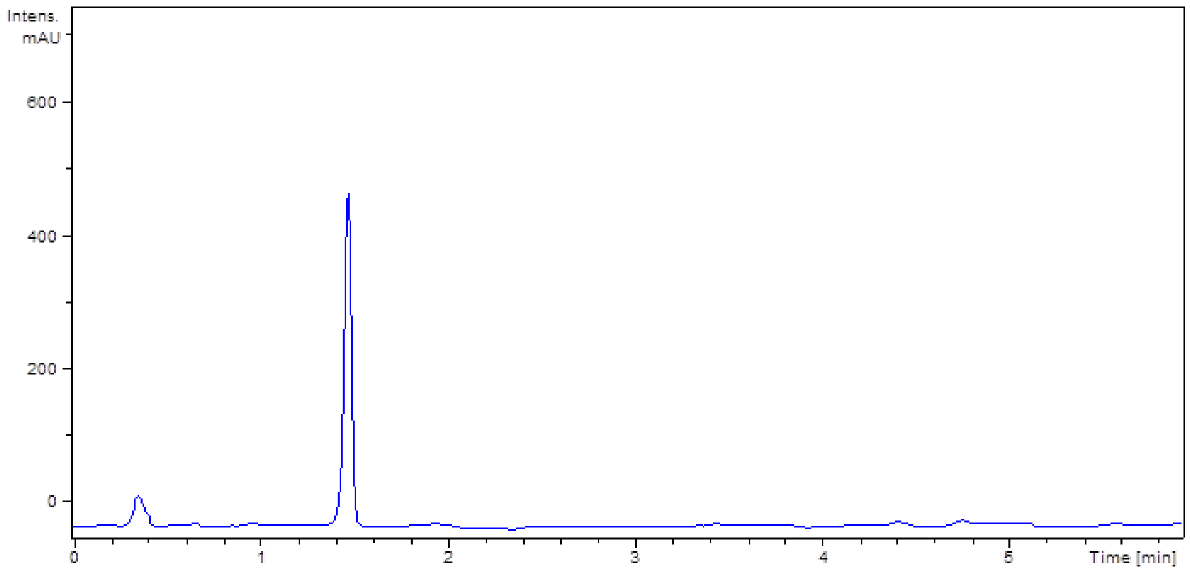

Compound 7a:

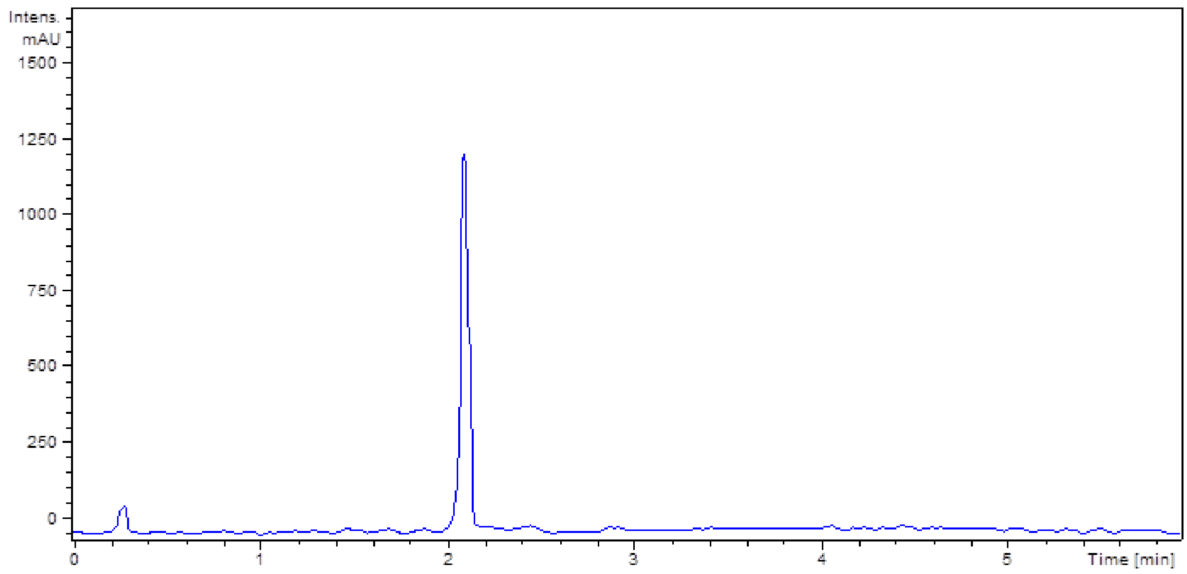

Compound 7b:

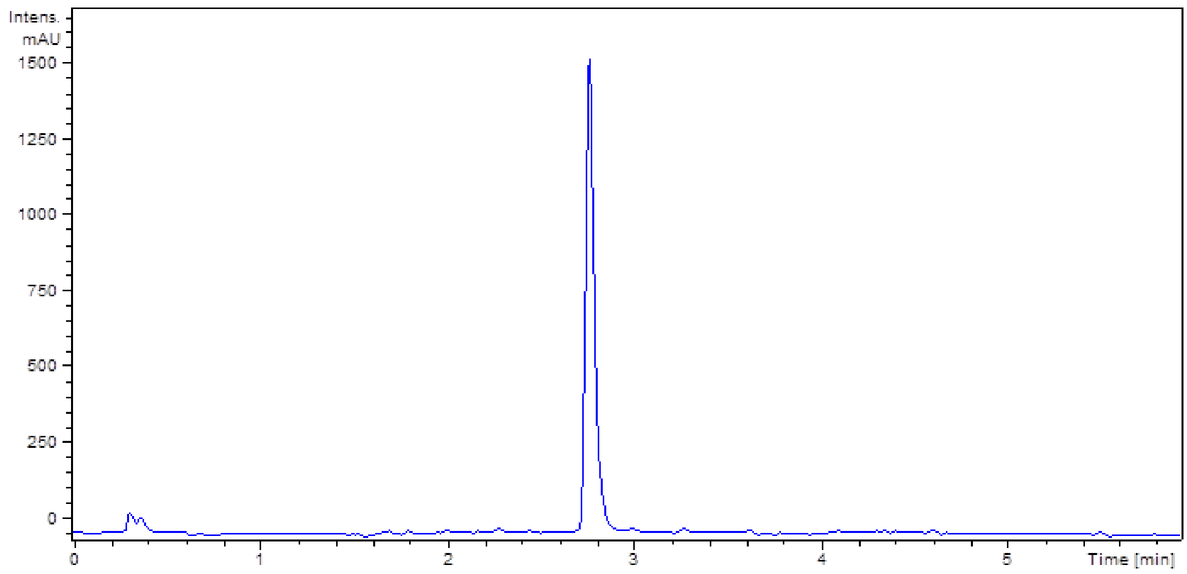

Compound 7c:

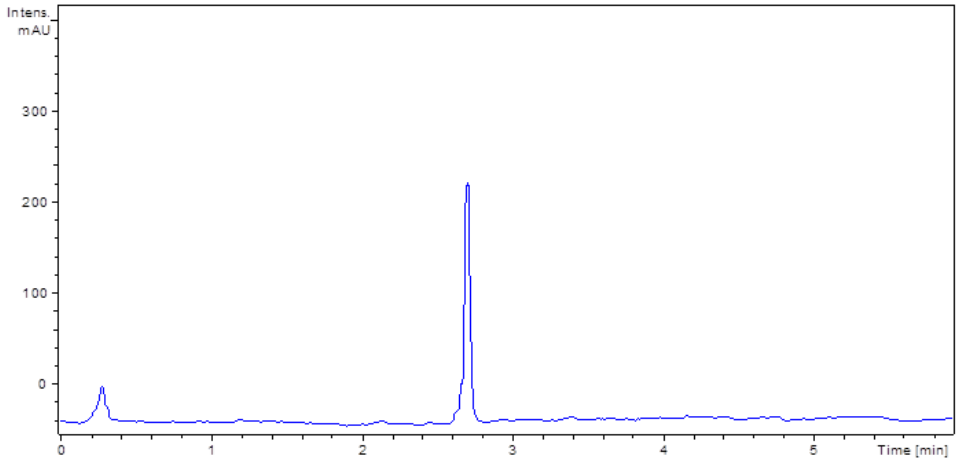

Compound 7d:

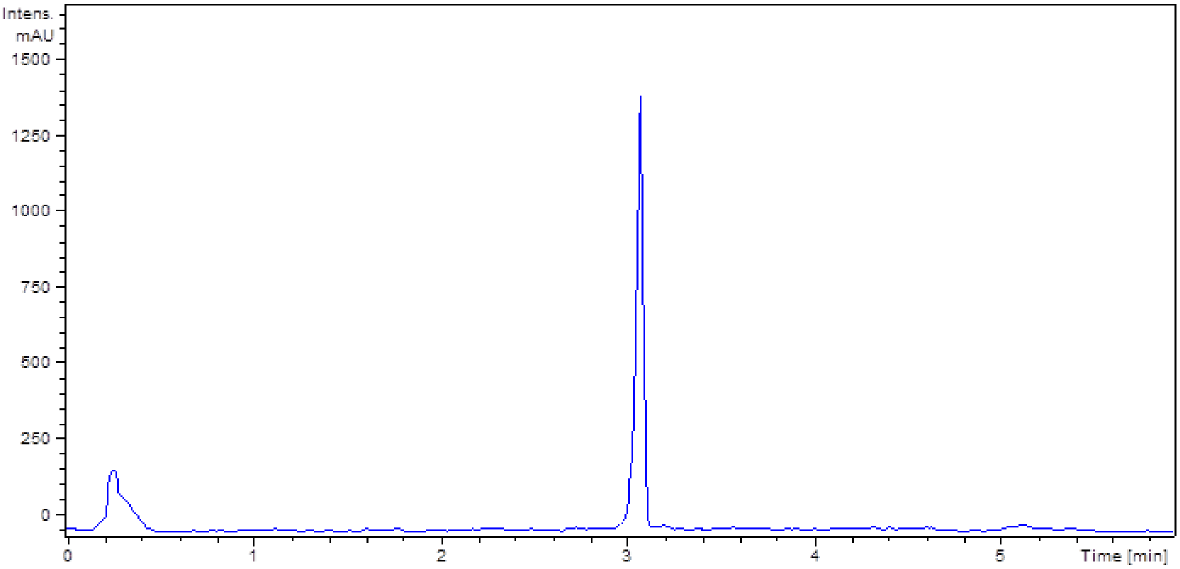

Compound 7e:

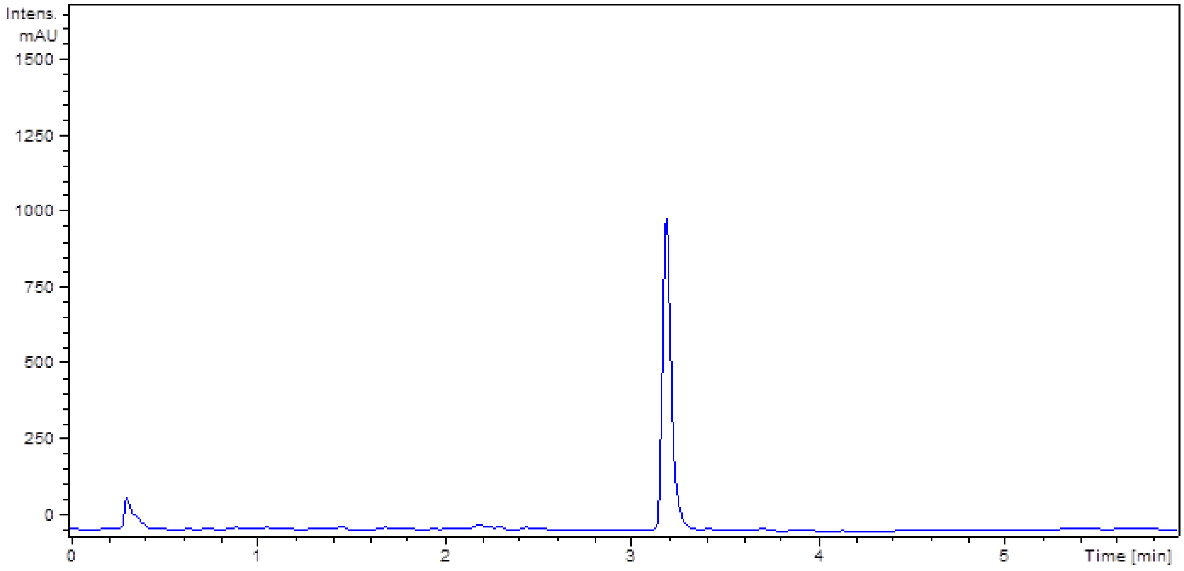

Compound 7f:

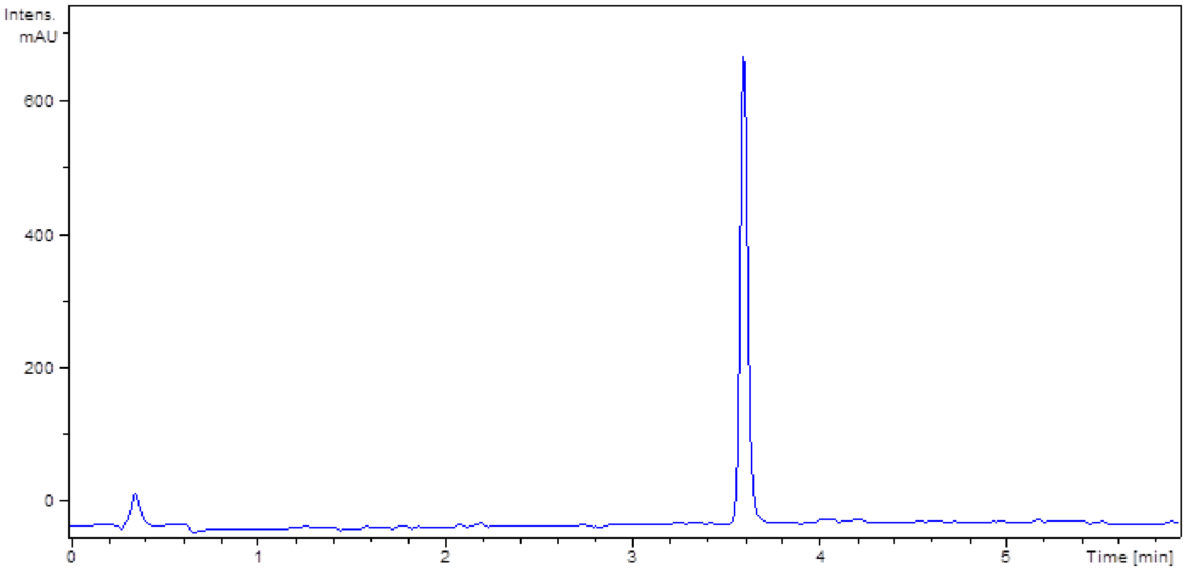

Supplement: Supplementary file 1 [file molecules-24-03959-s001.pdf]
